# The multidimensional randomized response design: Estimating different aspects of the same sensitive behavior

**DOI:** 10.3758/s13428-015-0583-2

**Published:** 2015-04-16

**Authors:** Maarten J. L. F. Cruyff, Ulf Böckenholt, Peter G. M. van der Heijden

**Affiliations:** Department of Methodology & Statistics, Utrecht University, Padualaan 14, 3584 CH Utrecht, The Netherlands; Kellogg School of Management, Northwestern University, Evanston, IL USA; S3RI, University of Southampton, Southampton, Great Britain

**Keywords:** Randomized response, Power, Efficiency, Response bias

## Abstract

The conventional randomized response design is unidimensional in the sense that it measures a single dimension of a sensitive attribute, like its prevalence, frequency, magnitude, or duration. This paper introduces a multidimensional design characterized by categorical questions that each measure a different aspect of the same sensitive attribute. The benefits of the multidimensional design are (*i*) a substantial gain in power and efficiency, and the potential to (*ii*) evaluate the goodness-of-fit of the model, and (*iii*) test hypotheses about evasive response biases in case of a misfit. The method is illustrated for a two-dimensional design measuring both the prevalence and the magnitude of social security fraud.

## Introduction

In surveys and questionnaires, sensitive questions are the major source of evasive responses. Respondents tend to avoid self-stigmatizing answers that reveal their true state as deviating from the norm. For example, if respondents believe their behavior is below or above average, they respectively over- or underreport their actual actions, ranging from exercise (Tourangeau et al. 1997) to income levels (Moore et al. 1999). Other examples of social desirability and acquiescence in self-reports on personal and sensitive issues are abound (Hurd 1999; Tourangeau & Yan, 2007, Van Soest & Hurd, 2008, Krumpal, 2012). To lower the incidence of evasive responses, recent work has focused on response–elicitation methods that protect the privacy of the respondent. One such method that has received considerable attention is randomized response. Under the randomized response design, the answer to a sensitive questions like “Did you use cocaine this month?” is determined by the combination of the respondent’s true state and the outcome of a randomizer. Since the outcome of the randomizer is known only to the respondent, the privacy of the respondent is protected and the risk of an evasive response is reduced.

The costs of privacy protection are two-fold. First, there is a loss of statistical efficiency; randomized response requires larger samples than direct questioning to obtain similar standard errors. To keep sample size requirements in check, there is a strong need to improve the efficiency of the randomized response design (e.g., Boruch, 1971; Fox & Tracy, 1986; Mangat, 1994; Gjestvang & Singh, 2006; Chaudhuri 2011; Moshagen, Musch, & Erdfelder, 2012). In spite of these efforts, it may still be problematic to significantly estimate a sensitive category with a prevalence close to zero. This is especially true for sensitive attributes with more than two categories, and this may very well be the reason that such highly informative polytomous questions as “How much cocaine did you use last month?” have been rarely used in randomized response. Second, although randomized response yields more valid answers than direct questioning (Lensvelt-Mulders et al. 2005), there is no guarantee that the privacy–protection mechanism completely eliminates evasive response behavior. As a consequence, prevalence estimates may be biased.

Thus, when choosing a randomized response design, one should not only consider its power but also its potential for detecting response biases. The unidimensional design is limited in this respect, because its statistical model is saturated. Consequently, it is expected to fit the data perfectly, even in the presence of response biases (Van den Hout and Van der Heijden 2004). This is not a satisfactory situation, and several multivariate models for the unidimensional design have been proposed that allow for the estimation of response biases. However, in order to identify these models, it is necessary to make strong distributional assumptions with respect to the sensitive attributes under investigation, such as a latent scale and conditional independence in IRT models (Bockenholt & Van der Heijden 2007; De Jong, Pieters, & Fox, 2010), the absence of the highest-factor interaction in a log-linear model (Cruyff et al. 2007), and the specification of informative priors in a Bayesian model (Van den Hout and Klugkist 2009). Notable exceptions are the unidimensional two-group models (Clark and Desharnais 1998; Moshagen et al. 2011; Van den Hout et al. 2010), which create degrees of freedom by dividing the sample into two groups with different randomization probabilities. These models, however, either leave the true state of the instruction non-compliant respondents unspecified (Clark and Desharnais 1998), or assume that non-carriers of the sensitive attribute always comply with the instructions (Moshagen et al. 2012). A major benefit of the multidimensional design is that such assumptions are either unnecessary or testable.

The key reason for proposing a multidimensional randomized response design is that it addresses the issues of efficiency and goodness-of-fit in a satisfactory way. A power study shows that the design is highly efficient, and therefore ideally suited for small prevalence estimation. In addition, the statistical model for multidimensional design has the desirable property of not being saturated. As a result, a meaningful goodness-of-fit test can be performed, and hypothesis testing of evasive responses is straightforward, and without any need to specify strong distributional assumptions to identify the model. These benefits are critical in sensitive question applications as well as in experimental studies that investigate conditions that trigger evasive responses.

The remainder of the paper is structured as follows. We first introduce a large-scale application on social security fraud (Lensvelt-Mulders et al. 2006) that allows illustrating the benefits of the proposed design. In “The model” section, we derive the statistical model for the multidimensional design, and in “Power study” section we present a power study comparing the uni- and multidimensional designs. Section “The analysis” returns to the data set and presents the results obtained with the proposed statistical models. We conclude the paper by summarizing the main results and by discussing future research topics in “Discussion” section.

## The data

At the turn of century, social security fraud was a much debated issue in the Netherlands. In 2000, the Dutch Department of Social Services started a program to monitor the prevalence of social security fraud. Every 2 years, a computer-assisted self-administered (CASI) survey was conducted with a unidimensional randomized response design characterized by a series of dichotomous questions assessing the prevalence of various types of social security fraud (see Lensvelt-Mulders et al., 2006). Since the department was also interested in the amounts of money involved, the survey was extended in 2004 with polytomous questions. We consider the following dichotomous and polytomous survey questions inquiring about the prevalence and magnitude of unreported income from odd jobs:

A: Have you ever in the past 12 months done small jobs for friends or acquaintances without reporting the income to the Department of Social Services?
B: On average, how much money a month (in euros) have you earned in the past 12 months in addition to your social security benefit by doing small jobs for friends or acquaintances without reporting this to the Department of Social Services?

Question A has response categories “No” and “Yes”, coded as 0 and 1, and question B has six response categories “0”, “1–50”, “51–100”, “101–150”, “151–250” and “more than 250”, coded 0 to 5. The responses to these questions were independently randomized according to the Forced Choice design (Boruch 1971): Respondents were instructed to throw two virtual dice and answer question A with “Yes” if the sum of the dice was 2, 3 or 4, with “No” if the sum of the dice was 11 or 12, and give a truthful answer if the sum of the dice was 5, 6, 7, 8, 9, or 10. To answer question B, the respondents threw another set of dice and answered truthfully if the outcome was 5, 6, 7, 8, 9, or 10. Otherwise, a single virtual die was thrown, and the number of eyes minus one was reported.

Under this randomization scheme, the transition probabilities denoting the probability of the observed answer given the respondent’s true state, are computed as the sum of a truthful and a forced responses. On question A, the probability of answering “Yes” given that the respondent enjoyed unreported income equals 3/4 + 1/6 = 11/12, and the probability of answering “No” given that the respondent did not enjoy unreported income is 3/4 + 1/12 = 5/6, where 3/4 is the probability of a truthful answer. On question B, the probability that the answer corresponds to the respondent’s true state equals 3/4 + 1/24 = 19/24, where 3/4 is again the probability of a truthful answer, and 1/24 = 1/4 × 1/6 is the probability of a forced response equal to the respondent’s true state.

During the course of the survey, however, it was discovered that a programmer inadvertently had programmed biased dice. After this was corrected, a total of 302 social security beneficiaries completed the survey using the unbiased dice. Table 1 presents the observed response frequencies *n*_*jk*_ for this group, with *jk* denoting the response profiles 00, … , 15.

**Table 1:** Observed response profile frequencies

*jk*	Response profile	*n*_*jk*_	*jk*	Response profile	*n*_*jk*_	total
00	“No, 0”	178	10	“Yes, 0”	25	203
01	“No, 1–50”	9	11	“Yes, 1–50”	29	38
02	“No, 51–100 ”	6	12	“Yes, 51–100”	9	15
03	“No, 101–150”	6	13	“Yes, 101–150”	10	16
04	“No, 151–250”	9	14	“Yes, 151–250”	12	21
05	“No, 250+”	5	15	“Yes, 250+”	4	9
Total		213			89	302

## The model

This section derives a statistical model for the analysis of categorical multidimensional randomized response data. Before introducing this approach, we review models for the analysis of categorical unidimensional data to clarify the key differences between the uni- and multidimensional approaches. We also discuss model estimation, the goodness-of-fit test, and the modeling of response biases.

### Unidimensional design analysis

The unidimensional design is characterized by one or more categorical questions—be it dichotomous or polytomous—that each addresses a different sensitive attribute. These questions may be analyzed either in a univariate or in a multivariate manner. A general model for the unidimensional design is

1 $$ P(R=r) = \sum\limits_{s}P(R=r|{S=s})P(S=s). $$

In the univariate version of this model, *R* is the variable denoting the *m* response categories of the sensitive question, *S* the variable denoting the *m* corresponding true states, and *P*(*R* = *r*|*S* = *s*) is the transition probability of observing response *r* given the true state *s*, for *r*, *s* ∈ {0, … , *m* − 1} and *m* ≥ 2. The transition probabilities can be derived from the known outcome distribution of the randomizer (Fox and Tracy 1986; Chaudhuri and Mukerjee 1988; Kim and Ward 2005).

In the multivariate version of the model, the variable *R* denotes the response profiles and *S* the corresponding true states profiles. For example, the bivariate model for two questions with *m* and *k* response categories has *m* × *k* response profiles and *m* × *k* corresponding true state profiles, as given by *s*, *r* ∈ {00, … , (*m* − 1)(*k* − 1)} for *m*, *k* ≥ 2. The transition probabilities for a multivariate model with *J* questions are obtained as

2 $$ P(R=r|{S=s}) = \prod\limits_{j=1}^{J} P(R_{j}=r_{j}|{S_{j}=s_{j}}), $$

where *P*(*R*_*j*_ = *r*_*j*_|*S*_*j*_ = *s*_*j*_) denotes the univariate transition probability for question *j* (Kuha and Skinner 1997).

### Multidimensional design analysis

The multidimensional design is characterized by multiple categorical questions addressing different dimensions of the same sensitive attribute. Since on each dimension the true states denoting zero intensity overlap, the multidimensional design with *J* questions has fewer true state profiles than the unidimensional designs with *J* questions. Without loss of generality, we illustrate this for the two-dimensional design.

Let the variables $R_{A_{1}}$ and $R_{A_{2}}$ denote the respective *m* and *k* response categories of two questions that each measure a different dimension of the same sensitive attribute. For example, the question “Did you use cocaine last month?” measures the dichotomous user/non-user dimension, while the question “How much cocaine did you last month?” measures a polytomous quantity dimension. Obviously, when one has not used cocaine, one cannot have used a positive amount. Vice versa, when one has used cocaine, one cannot have used a zero amount. It follows that only the 1 + (*m* − 1)(*k* − 1) true states combinations $S_{A_{1}}=0,S_{A_{2}}=0$ and $S_{A_{1}}>0,S_{A_{2}}>0$ can occur. However, due to the randomization of the responses all *m* × *k* combinations of the response categories of $R_{A_{1}}$ and $R_{A_{2}}$ can occur. Hence, the model for the two-dimensional design is

3 $$ P(R=r) = \sum\limits_{s^{*}}P(R=r|{S^{*}=s^{*}})P(S^{*}=s^{*}), $$

where *R* ∈ {00, … , (*m* − 1)(*k* − 1)} denotes the full set of response profiles, and *S*^∗^ ∈ {00, 11, … , (*m* − 1)(*k* − 1)} the reduced set of feasible true state profiles. The two-dimensional transition probabilities *P*(*R* = *r*|*S*^∗^ = *s*^∗^) following from expression (2) after substitution of *S* for *S*^∗^. The model has *m* + *k* − 2 degrees of freedom, which correspond to the number of true state profiles that cannot occur.

### Estimation and goodness-of-fit

The maximum likelihood estimator (MLE) of the true state probability vector ***π*** under the multidimensional design can be obtained by maximizing the log-likelihood function

4 $$ \ln\ell(\boldsymbol{\pi}|\boldsymbol{n}) = \sum\limits_{r}n_{r}\ln(p_{r|s^{*}}\pi_{s^{*}}), $$

where ***n*** is the vector of the observed frequencies *n*_*r*_ of response profiles *r*, $p_{r|s^{*}}$ is shorthand notation for *P*(*R* = *r*|*S*^∗^ = *s*^∗^), and $\pi _{s^{*}}$ the prevalence of true state profile *s*^∗^ (for the unidimensional design, the notation is with the subscripts *s* instead of *s*^∗^). The R code for model estimation is given in Appendix B.

A commonly used goodness-of-fit test for models with categorical data is the likelihood-ratio test statistic

5 $$ G^{2}=2\sum\limits_{r}n_{r}\ln(n_{r}/\hat{n}_{r}), $$

where *n*_*r*_ and $\hat {n}_{r}$ denote the respective observed and fitted response frequencies (using the convention 0⋅ ln(0) = 0 in case *n*_*r*_ = 0). For sufficiently large *n*, the *G*^2^-statistic is approximately Chi-squared distributed with the degrees of freedom equal to the number of response profiles minus the number of true state profiles. Under the unidimensional design, where the number of responses profiles equals the number of true state profiles, the *G*^2^-statistic is expected to be zero, but this need not be the case. This awkward result occurs when an observed frequency is below the minimum expected frequency, i.e., the frequency expected when the prevalence of the corresponding true state is zero. Reasons for this to happen are sampling error (in the outcomes of the randomizer) and/or instruction noncompliance, such as the presence of evasive responses in the data (Van den Hout and Van der Heijden 2004). These effects are confounded, but since there is no theory for a positive *G*^2^-statistic on zero degrees of freedom, the *G*^2^-test cannot be used to test for the presence of evasive response biases. Under the multidimensional design, however, the model has the necessary degrees of freedom to perform a meaningful goodness-of-fit test. If the test is non-significant, the misfit can be attributed to sampling error, but a significant test provides evidence for the presence of response biases. In the latter case, the degrees of freedom can also be utilized to test hypotheses about the nature of the response biases, which may help us to better understand the psychological mechanisms that lead to evasive response behavior. Below we present two such response bias models for the two-dimensional design.

### Tests for response biases

The response bias models we consider assume two rather different psychological mechanisms that may lead to response biases. The first mechanism assumes that a response bias is a question effect in the sense that the sensitivity of a question may elicit response biases. The second mechanism assumes that a response bias is caused by person effects in the sense that some respondents may answer questions evasively.

Consider two questions A and B that trigger evasive responses with respective probabilities *θ*_*A*_ and *θ*_*B*_, independently of a person’s true state. In this case, the transition probabilities of observing a non-zero response to question A are decreased by a factor 1 − *θ*_*A*_, and those for question B by a factor 1 − *θ*_*B*_. The modified transition probabilities for both questions A and B thus become

6 $$\begin{array}{@{}rcl@{}} \tilde{P}(R_{j}&=&r_{j}|S_{j}=s_{j}) = (1-\theta_{j}){P}(R_{j}=r_{j}|S_{j}=s_{j})\\ &&+I(R_{j}=0)\theta_{j}, \end{array} $$

where *j* ∈ {*A*, *B*} is an index for the question and *I*(*R*_*j*_ = 0) an indicator variable taking value 1 if the observed response is “0”. These probabilities are plugged into expression (3) to obtain the transition probabilities for the question–effect model.

Under the person–effect model, we assume that a subset of respondents answers both questions evasively, irrespectively of their true state or the outcome of the dice. For this model, the transition probabilities are obtained as

7 $$\begin{array}{@{}rcl@{}} \tilde{P}(R=r|S^{*}=s^{*})&=&(1-\theta){P}(R=r|S^{*}=s^{*})\\&&+I(R=00)\theta, \end{array} $$

where *P*(*R* = *r*|*S*^∗^ = *s*^∗^) are the transition probabilities of the basic two-dimensional model, *θ* is the prevalence of the class of evasive respondents, and *I*(*R* = 00) an indicator taking value 1 if the observed response profile is “00”. Estimation of these models follows the same principles as described in the previous section (for the R code we refer to Appendix B).

Both models are applied in “The analysis” section to demonstrate the capabilities of the multidimensional design in testing for response biases. We do note, however, that the two hypotheses on person and item effects are by no means exhaustive. For example, more complex hypotheses might consider the true state of the respondent, or the possibility of non–zero evasive responses to the polytomous question. Given the uncertainty about the validity of the assumptions, we stress caution in interpreting the results obtained by these models. We suggest to apply these models as part of a sensitivity analysis that evaluates the robustness of the original model against the presence of evasive responses. If these tests point to the presence of evasive responses, additional studies are needed to examine and validate the underlying response bias mechanism.

## Power study

This section presents the main results of this paper by comparing the power and efficiency of four unidimensional designs and one multidimensional design. Three of the unidimensional designs are dichotomous. The least efficient of these is the one by Warner (1965), which involves the randomization of the sensitive question and its complement. A more efficient design is Forced Choice (FC2) by Boruch (1971), which involves the randomization of truthful and forced responses. The study also includes a trichotomous version (FC3). One of the most efficient unidimensional designs at present is the one by Mangat (1994), and is comparable to the FC2 design with the difference that respondents with the sensitive attribute are expected to answer the question truthfully. As the representative for the multidimensional design, we have selected the two-dimensional Forced Choice design with a dichotomous and trichotomous question, which is labeled as FC2x3 (Table 2).

**Table 2:** Transition probabilities of the uni- and two-dimensional design(s)

	Warner	FC2	Mangat	FC3	FC2x3
*p*_0|0_	5/6	5/6	5/6	5/6	25/36
*p*_1|1_	5/6	11/12	1	5/6	55/72
*p*_2|2_	–	–	–	5/6	55/72

The efficiency of the unidimensional designs in this study can be compared on the basis of their transition probabilities. For comparability, the transition probabilities *p*_0|0_ are set to 5/6 for all four designs. For the (dichotomous) Warner, FC2 and Mangat designs, *p*_1|1_ is respectively set to 5/6, 11/12, and 1. For the FC3 design, *p*_*r*|*s*_ is set to 5/6 for all *r* = *s*. The transition probabilities of FC2x3 design are derived according to Eq. 2 using the univariate transition probabilities of FC2 and FC3.

**Fig. 1:**
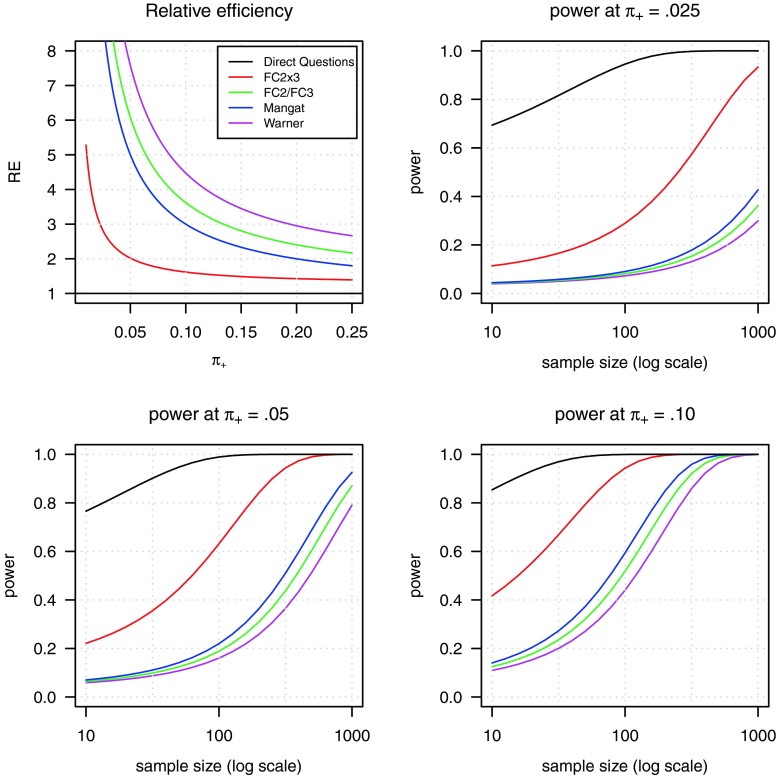
RE and power curves for the estimator of *π*_+_

The top-left panel of Fig. 1 shows the efficiency of the various randomized response designs relative to that of the direct question design for *π*_+_ = 1 − *π*_0_ ∈ {0, .25}. The relative efficiency (RE) of the estimator for *π*_+_ is computed as

8 $$\begin{array}{@{}rcl@{}} RE(\hat\pi_{+}) = \frac{\text{var}_{_{RR}}(\hat\pi_{+})}{\text{var}_{_{DQ}}(\hat\pi_{+})}, \end{array} $$

with $\text {var}(\hat {\pi }_{+}) = \text {var}(1-\hat {\pi }_{0}) = \text {var}(\hat {\pi }_{0})$ denoting the analytical sampling variance (for the derivations of the analytical sampling variances, we refer to Appendix 1). The RE-curves show that the two-dimensional FC2x3 design is substantially more efficient than all four unidimensional designs. For example, for *π*_+_ = .05, the former design needs to increase the sample size by a factor to obtain the same precision as the direct question design, while the unidimensional designs require factors ranging between 5 and 8.

The remaining three panels of Fig. 1 display the power curves of the various designs. The power is defined as the probability of rejecting the null hypothesis *H*_0_ : *π*_+_ = *μ*_0_ given that the alternative *H*_*A*_ : *π*_+_ = *μ*_*A*_ is true, and is computed as

9 $$ \gamma={\Phi}\left(\frac{\mu_{A}-\mu_{0}+z_{\alpha}\cdot\sigma_{0}}{\sigma_{A}}\right), $$

where *σ*_0_ and *σ*_*A*_ are the respective standard deviations of *π*_+_ under *H*_0_ : *π*_+_ = 0 and *H*_*A*_ : *π*_+_ ∈ {.025, .05, .10}, and *z*_*α*_ the *z*-score at significance level *α* (Ulrich et al. 2012). The top-right panel shows that, for the alternative that *π*_+_ = .025, the FC2x3) attains a power of 80 % with *n* ≈ 630, while for that sample size the unidimensional designs have a power of 15 % or less. For the alternative that *π*_+_ = .05, the FC2x3 requires *n* ≈ 180 to attain 80 % power, while the unidimensional designs need between 600 and 850 respondents. For the alternative *π*_+_ = .10 *%*, the FC2x3 attains 80 % power at *n* ≈ 50, while the unidimensional designs require sample sizes between 200 and 250 respondents.

## The analysis

We analyzed the social security survey data in “The data” section with unidimensional FC2 and FC6 models, and the two-dimensional FC2x6 design. Table 3 reports the prevalence estimates and (analytical) standard errors of the three models. Since the models FC6 and FC2x6 yield boundary estimates, the analytical standard errors may be biased. Consequently, a parametric bootstrap was conducted to obtain empirical standard errors, reported in the table as se _*B*_.

**Table 3:** Prevalence estimates and goodness-of-fit tests

	FC2		FC6		FC2x6	
	Est.	SE	Est.	SE (SE_*B*_)	Est.	SE (SE_*B*_)
*π*_0_	82.9	3.5	83.0	3.6 (3.4)	79.7	2.7 (2.7)
*π*_1_	17.1	3.5	11.0	2.5 (2.6)	11.7	2.3 (2.3)
*π*_2_			1.0	1.7 (1.3)	2.2	1.4 (1.3)
*π*_3_			1.4	1.7 (1.4)	2.7	1.4 (1.4)
*π*_4_			3.6	1.9 (1.9)	3.7	1.6 (1.6)
*π*_5_			0.0	1.5 (0.0)	0.0	0.9 (0.0)
	$G^{2}_{(0)}=0$		$G^{2}_{(0)}=1.8$		$G^{2}_{(6)}=9.3$	

The models FC2 and FC6 for the unidimensional designs yield prevalence estimates of unreported income fraud of 17.1 % and 17.0 %, with 95 % confidence intervals (10.2, 23.9) and (9.9, 24.1) (based on the analytical standard errors). The FC2x6 model for the two-dimensional design yields a slightly higher estimate of 20.3 %, with the 95 % confidence interval (14.9, 25.6). Comparing the standard errors of the models, we find that the FC2x6 model yields the most precise estimates. Furthermore, the FC6 model obtains only one significant estimate for the positive amounts of unreported income, while the FC2x6 model yields three (with the additional estimate $\hat \pi _{3}$ being on the verge of significance). Obviously, the FC2x6 model allows for more confident conclusions about the distribution of unreported income than the FC6 model. Aside from the boundary estimate $\hat {\pi }_{5}$, the analytical standard errors are close to the bootstrap standard errors (especially for the FC2x6), so that the quality of the former appear satisfactory.

When evaluating the fit of the models, we see that the FC2 model fits as expected, but that model FC6 has a non-zero *G*^2^-statistic. This result is due to the observed response frequency *n*_5_ = 9, which is smaller than the expected minimum *n*⋅*P*(*R* = forced “5”) = 302/24 = 12.6. Since the model does not have any degrees of freedom, we cannot perform a goodness-of-fit test or fit a response–bias model. Consequently, we cannot determine the likelihood of evasive responses in the data, or examine the nature and prevalence of potential response biases. However, for the FC2x6 model, such tests can be performed. The goodness-of-fit test *G*^2^ = 9.3, *df* = 6, *p* = .16) indicates that the fit of the model is satisfactory. We note that the presence of response biases cannot be ruled out; the goodness-of-fit test may not have enough power to detect response biases and the *G*^2^ value leaves room for improvement. To learn more about the potential presence of response biases in the data, we fit the two response bias models described in the model section.

The question–effect model does not affect the fit at all, but the person–effect model significantly reduces the *G*^2^ value with 8.3 points on one degree of freedom *G*^2^ = 1.0, *df* = 5, *p* = 0.96). Its estimate $\hat {\theta }=0.217$ and the corrected prevalence estimates (with 0.001 rounding error) $\hat {\boldsymbol {\pi }}=$(0.719, 0.157, 0.032, 0.038, 0.053, 0.000) imply that $\hat {\theta }(n-n_{00})/n\approx 9~\%$ of the observed response profiles is actually misreported as “00”. Although this model is significant, there is a risk of overfitting and uncertainty about the validity the model assumptions. Therefore we interpret the results as a worst-case scenario, and the corrected prevalence estimate of 28.3 % as the upper bound for unreported income. Thus, ignoring evasive response biases in our original model (with the prevalence estimate of 20.3 %) may bias the prevalence estimate of unreported income by as much as 8 %.

The boundary solution, in combination with a relatively small sample and the flexibility of the person–effect model (5 degrees of freedom for fitting 12 response frequencies) may have compromised the consistency of the estimates and may have resulted in low power to detect the person effect. To investigate this, we performed a parametric bootstrap by generating 2 × 10,000 random samples of respective sizes *n* = 302 and *n* = 1,000 from a population with parameters *θ* and ***π*** as estimated for the social security data. We applied the randomized response procedure and the person effect to these samples, and fitted the person–effect model. Table 4 reports the averaged bootstrap estimates, the 95 % confidence intervals, and the power estimates (the proportion of samples with a significant estimate $\hat {\theta }$).

**Table 4:** Bootstrap estimates for the person–effect model

	*n* = 302		*n* = 1000
	Value	Est. (95 % CI)	Est. (95 % CI)
*π*_0_	0.719	0.716 (0.617; 0.798)	0.718 (0.666; 0.766)
*π*_1_	0.157	0.158 (0.095; 0.229)	0.157 (0.122; 0.195)
*π*_2_	0.032	0.033 (0.002; 0.071)	0.032 (0.014; 0.052)
*π*_3_	0.038	0.039 (0.005; 0.080)	0.038 (0.019; 0.059)
*π*_4_	0.053	0.053 (0.015; 0.099)	0.053 (0.032; 0.077)
*π*_5_	0.000	0.002 (0.000; 0.025)	0.001 (0.000; 0.013)
*θ*	0.217	0.219 (0.076; 0.354)	0.217 (0.142; 0.292)
Power	84.5 %		99.9 %

The results in Table 4 show that the parameter estimates are minimally biased for *n* = 302, and that this bias disappears almost completely as the sample size increases. Importantly, the power to detect the person effect is more than adequate for sample size of 302, and approaches 1 as the sample size increases to 1,000.

## Discussion

This paper introduced a multidimensional design for randomized response studies. We showed that this design has three major advantages over current unidimensional randomized response design. It enables prevalence estimation of the various levels of a sensitive attribute with significantly higher precision. Furthermore, it allows for model evaluation, and in case of misfit, for testing hypotheses about the presence of evasive response biases. These are important benefits that should facilitate the application of randomized response designs. We illustrated these benefits for Boruch’s (1971) FC approach, but modifications to accommodate other randomized response designs are straightforward and require few changes.

A frequently used strategy for demonstrating the usefulness of randomized response methods is to compare the estimated prevalence rates to the ones obtained by asking respondents directly. Our analyses show that this comparison can lead to incorrect conclusions. When respondents do not follow the design, the resulting prevalence estimates may be severely biased. A more informative and behaviorally descriptive approach is to test for the presence of different forms of misreporting. This provides both insight in the robustness of our prevalence estimates against misreporting and in the likelihood of different evasive response mechanisms. Validation of these mechanisms in subsequent studies will improve our knowledge about evasive response behavior in randomized response settings. The proposed statistical model for the multidimensional randomized response design provides an easy-to-implement approach for this purpose.

In addition to efficiency and goodness-of-fit considerations in comparing randomized response designs, it may also be valuable to investigate the degree to which they make respondents feel protected under various randomized response designs. Randomized-response designs with the same statistical properties (i.e., identical transition probabilities) may not have the same psychological properties. The degree to which different randomized response designs, as those by Warner (1965), Greenberg et al. (1969), Boruch (1971) and Kuk (1990), trigger misreporting would thus be a fruitful area of research. To illustrate this, consider a Warner and FC design with identical transition probabilities *p*_0|0_ = *p*_1|1_ = 0.8. Under the FC design, respondents without the sensitive attribute have a 20 % chance to be forced to (incorrectly) answer “Yes” to the sensitive question, while under the Warner design they have a 20 % chance to (correctly) answer “Yes” to the complementary question. The latter randomization mechanism may be perceived as less intrusive, and therefore provoke fewer evasive responses. Clearly, experimental investigations of these designs are needed that improve our understanding of the psychological conditions that lead to evasive responses so that they can be controlled for effectively. Given its capacities for modeling response biases, the multidimensional design would be well suited for this purpose.

## Appendix A: Sampling variance of $\hat {\boldsymbol {\pi }}_{+}$

Section A of the appendix presents the analytical sampling variances for unidimensional and two-dimensional designs of Section 3.

For the dichotomous unidimensional designs, the sampling variance of the prevalence estimate $\hat {\pi }_{1}=\hat {\pi }_{+}$ is most easily derived by the method of moments. To simplify notation, let *π*_*s*_ = *P*(*S* = *s*), $\pi ^{*}_{r}=P(R=r)$, and *p*_*r*|*s*_ = *P*(*R* = *r*|*S* = *s*). The sampling variance of the response probability $\pi ^{*}_{1}$ is

10 $$ \text{var}(\hat{\pi}^{*}_{1}) = \frac{\pi^{*}_{1}(1-\pi^{*}_{1})}{n}, $$

where $\pi ^{*}_{1}$ is the probability of a “1” response in the sample. Since model (1) follows that ${\pi }^{*}_{1}=(p_{1|1}-p_{1|0}){\pi }_{1}+p_{1|0}$, the moment estimator of *π*_1_ can be derived as

11 $$ \hat{\pi}_{1}=\frac{\pi^{*}_{1}-p_{1|0}}{p_{1|1}-p_{1|0}}, $$

where $\pi ^{*}_{1}$ is estimated as the observed proportion of “Yes” responses in the sample. Since $n\hat \pi ^{*}_{1}$ has a binomial distribution $B(n,\pi ^{*}_{1})$, the sampling variance of *π*_1_ is given by

12 $$ \text{var}(\hat{\pi}_{1}) = \frac{\pi^{*}_{1}(1-\pi^{*}_{1})} {n(p_{1|1}-p_{1|0})^{2}}, $$

Kuk (1990) and Su et al. (2014). Notice that in Eq. 11 the estimate $\hat {\pi }_{1}$ can take a negative value if *n*_1_/*n* < *p*_1|0_. Since the moment estimator of *π*_1_ is not bounded by 0, its sampling distribution is asymptotically normal (Ulrich et al. 2012).

For polytomous unidimensional designs, the computation of the sampling variance of $\hat \pi _{1}$ involves covariances, and therefore the maximum likelihood method is better suited. The sampling variance of $\hat \pi _{1}$ can be obtained from the inverse of the Fisher information

13 $$ \mathbf{I}=-\mathrm{E}\left(\frac{\partial^{2}\ln\ell(\boldsymbol{\pi}|\boldsymbol{n})} {\partial\pi_{u}\partial\pi_{v}}\right), $$

with entries (*u* + 1, *v* + 1), for *u*, *v* ∈ {0, … , *m* − 1}, where *m* is the number of response categories. A difference with the method of moments is that prevalence estimates cannot be negative, so that the normal approximation may be compromised if there are estimates on the boundary of the parameter space.

The method is illustrated for the trichotomous FC3 design. To further simplify notation, let *d* denote the probability of a truthful response, and *e* = (1 − *d*)/3 the probability of a forced response. After some algebra, model (1) can be written as $\pi ^{*}_{s}=d\pi _{s}+e$, for *s* ∈ {0, 1, 2}. By defining ***π*** = (*π*_0_, *π*_1_,1 − *π*_0_ − *π*_1_), and plugging this vector in the log-likelihood (4), we obtain

14 $$\begin{array}{@{}rcl@{}} \sum\limits_{r}n_{r}\ln(\pi^{*}_{r})&=&n_{0}\ln(d\pi_{0}+e)+n_{1}\ln(d\pi_{1}+e)\\ &&+n_{2}\ln(d(1-\pi_{0}-\pi_{1})+e) \end{array} $$

Taking the partial derivatives with respect to *π*_0_ and *π*_1_, we obtain

15 $$\begin{array}{@{}rcl@{}} \frac{\partial^{2}{\sum}_{r}n_{r}\ln(\pi^{*}_{r})} {\partial\pi_{u}\partial\pi_{v}}= \left\{\begin{array}{ll} -n_{0}(d/\pi^{*}_{0})^{2}- n_{2}(d/\pi^{*}_{2})^{2}&\text{for }\; u=v=0\\\\ -n_{2}(d/\pi^{*}_{2})^{2}&\text{for }\; u\neq{v}\\\\ -n_{1}(d/\pi^{*}_{1})^{2} -n_{2}(d/\pi^{*}_{2})^{2}&\text{for }\; u=v=1 \end{array}\right. \end{array} $$

which, after taking the expectation $E(n_{r}) = n{\pi }^{*}_{r}$ and changing the sign, yields the Fisher information

16 $$\begin{array}{@{}rcl@{}} \textbf{I}=nd^{2}\left(\begin{array}{cc} 1/\pi^{*}_{0}+1/\pi^{*}_{2}&1/\pi^{*}_{2}\\\\ 1/\pi^{*}_{2} &1/\pi^{*}_{1}+1/\pi^{*}_{2} \end{array}\right) \end{array} $$

as defined in Eq. 13. Inversion of this matrix yields the variance-covariance matrix

17 $$\begin{array}{@{}rcl@{}} \frac{1}{nd^{2}}\left(\frac{1}{\pi^{*}_{0}\pi^{*}_{1}}+\frac{1}{\pi^{*}_{0}\pi^{*}_{2}} +\frac{1}{\pi^{*}_{1}\pi^{*}_{2}}\right)^{-1} \left(\begin{array}{cc} 1/\pi^{*}_{1}+1/\pi^{*}_{2}&-1/\pi^{*}_{2}\\\\ -1/\pi^{*}_{2}&1/\pi^{*}_{0}+1/\pi^{*}_{2} \end{array}\right), \end{array} $$

with the sampling variances of *π*_0_ and *π*_1_ on the diagonal, and the sampling variance of *π*_2_ given by the sum of the elements in Eq. 17. To obtain the sampling variance of *π*_+_, we note that $\text {var}(\hat \pi _{+}) = \text {var}(1-\hat \pi _{0}) = \text {var}(\hat \pi _{0})$. The Fisher information for the two-dimensional FC designs is derived analogously, but with substantially more tedious algebra. The analytical expressions for its inverse are intractable, so that the inversion is best performed numerically with the help of a computer program.

## Appendix B: R-code for model estimation

Section B of the appendix presents R code for the estimation of the two-dimensional model for the social security data. For efficient programming the summations in Eqs 2, 3, and 4 are computed using matrix algebra, and to ensure that the prevalence estimates stay within the boundaries of the parameter space, we use a logistic transformation.
